# An interventional study of baicalin on neuronal pentraxin-1, neuronal pentraxin-2, and C-reactive protein in Alzheimer’s disease rat model

**DOI:** 10.1515/tnsci-2022-0298

**Published:** 2023-09-06

**Authors:** Jing-Kun Zhao, Si-Jia Hou, Ji-Wei Zhao, Hong-Li Yu, Shu-Rong Duan

**Affiliations:** Department of Neurology, The First Affiliated Hospital of Harbin Medical University, No. 23 Postal Street, Nangang District, Harbin 150001, China; Department of Neurology, The General Hospital of Heilongjiang Province Land Reclamation Bureau, Harbin 150088, China

**Keywords:** baicalin, Alzheimer’s disease, neuronal pentraxin-1, neuronal pentraxin-2, C-reactive protein, rat model

## Abstract

**Background:**

Baicalin has been shown to promote spatial learning and neural regeneration, which might increase the differentiation of neural stem cells in Alzheimer’s disease (AD) rat models. We aimed to study the role of baicalin on neuronal pentraxin-1 (NPTX-1), neuronal pentraxin-2 (NPTX-2), and C-reactive protein (CRP) in AD model rats.

**Methods:**

The 30 male Sprague Dawley rats were divided into three groups: the control group, the AD model group, and the AD + baicalin group. Then, the Morris water maze was used to verify the effect of baicalin on the memory and spatial learning of rats. Immunohistochemistry and immunofluorescence were used to observe the expression of NPTX-1, NPTX-2, and CRP in brain tissue.

**Results:**

Compared with the AD model group, the AD rats treated with baicalin spent significantly less time finding escape latencies (*P* = 0.008) and had longer cross-platform times in the target quadrant (*P* = 0.015). In addition, the AD + baicalin group had significantly higher numbers of hippocampal neurons compared with the AD model group (*P* < 0.05). Baicalin also obviously decreased the apoptosis of neurons. Moreover, compared with the AD model group, the NPTX-1 and CRP expression in the AD + baicalin group was significantly reduced (*P* = 0.000) while the expression of NPTX-2 in the brain tissue of AD rats was significantly increased (*P* = 0.000).

**Conclusions:**

Baicalin can play a therapeutic role by downregulating NPTX-1, upregulating NPTX-2, and downregulating CPR in AD model rats.

## 1 Introduction

Alzheimer’s disease (AD) is a progressive neurodegenerative disorder with characteristic neuropathological marker of AD is extracellular β amyloid (Aβ) protein deposits, and nerve fiber tangles formed by phosphorylated Tau protein in cells. It has been reported that immunological changes, oxidative stress, microvascular changes, inflammatory reactions, and excitotoxicity were the main mechanisms in the development of AD [1]. Although much improvement has been made in treating AD, memory loss in AD remains an important unknown factor affecting the development of treatment [2]. The reduction in synapse numbers and the levels of soluble oligomeric forms of Aβ protein were the main cause of memory loss in AD [3,4]. Recently, researchers have focused on finding biomarkers related to AD, which will help for clinical treatment decision.

The prominent role of pentraxins (PTXs), including neuronal pentraxin-1 (NPTX-1), neuronal pentraxin-2 (NPTX-2), and C-reactive protein (CRP) in synaptic plasticity raises the possibility that its expression may be altered in AD that are characterized by synaptic dysfunction and loss [5]. Previous studies have indicated that NPTXs is the key factors of synaptic loss, ganglion damage, and neuronal death in AD induced by Aβ [6,7]. Besides, PTXs have been implicated in the regulation of glutamate neurotransmission in excitatory neurons. In synapses, NPTX1, NPTX2 combined with their receptor to recruit and cluster AMPA-type glutamate receptors [8]. NPTX1, NPTX2, and CRP were the key biomarkers for improving memory for AD.

Recently, baicalin has been shown to promote spatial learning and neural regeneration [9], which might increase the differentiation of neural stem cells in AD rat models [10]. Baicalin, which is also known as 7-glucuronic acid and 5, 6-dihydroxyflavone, is extracted from the dried roots of *Scutellaria baicalensis* Georgi. [11]. Jin et al. [12] indicated that baicalin is a potential neuroprotective compound for preventing and treating microglia-mediated neuroinflammation during AD progression. Yin et al. [13] showed that baicalin may inhibit Aβ aggregation and thereby delay or modify the progression of AD. Li et al. has suggested that baicalin could relieve oxidative stress damage in hippocampal neuron cells induced by glutamate [14]. NPTX1, NPTX2, and CRP were biomarkers related to neurochemical and glutamate, which might be a novel mechanism of treating AD by baicalin.

Thus, in our study, we evaluated the effect of baicalin on AD rats using Morris water maze (MWM). Then, the immunohistochemistry and immunofluorescence were used to observe the expression of NPTX-1, NPTX-2, and CRP in brain tissue. Our results may offer a theoretical basis for the use of baicalin, the active components of which promote nerve regeneration.

## 2 Materials and methods

### 2.1 Experimental drug

Baicalin (purity >99%) was supplied by Shanghai yuanye Bio-Technology Co., Ltd (Shanghai, China). Amyloid β-protein, Fragment1-40 (Aβ1-40) (Abcam, St Louis, MO, USA) was attenuated to 5 g/L with sterile double-distilled water, and then incubated in an incubator (37°C) for 7 days. After that, Aβ1-40 was stored at −20°C.

### 2.2 Animals and AD rats model preparation

A total of 30 healthy Sprague Dawley (SD) male rats (weighing 250–300 g) were obtained from the Animal Center of First Hospital affiliated to Harbin Medical University (Harbin, China). The SD rats were raised under the barrier system of room temperature and humidity (40–50%), and experimental conditions were kept according to the SPF standard.

The 30 SD rats were randomly classified into the control group, the AD model group, and the AD + baicalin group (*n* = 10 per group) for anesthesia without signs of peritonitis. Rat brains were fixed on a stereoscopic instrument, and the hair and scalp were removed to make the cranium expose. The hippocampus was injected unilaterally with 2 μL (10 µg) Aβ1-40 solution using a micro-syringe in 5 min. The control group was treated with an equal volume of sterile normal saline. After surgery, dental clay was used to seal the holes and the scalp was sutured and treated with a disinfectant to avoid infection [10]. The rats were kept in a single cage until fully awake. After obtaining the AD model, the AD + baicalin group was given 10 mg/kg of the drug via gavage for 30 days. The control group and the AD group were given an equal volume of saline via gavage.

### 2.3 MWM

The MWM (sansbio, China), was first applied by Morris (1984) to assess memory function and spatial learning which normally used visual cues [15]. We used the MWM to perform the place navigation test as follows. The rats were first placed into the water in southeast, northeast, northwest, and southwest, randomly [16]. The experimental device could track the movement of the rats in the water through the video system. The time from initial entry into the water to their first arrival at the platform was recorded as the escape latency. If the rats did not find the platform within 90 s, the rats were guided to the platform to rest for 15 s and the incubation period was recorded as 90 s. After 60 s, we proceeded to the next test. The rats were tested four times a day for 6 days, and the escape incubation period was recorded in detail using the video system.

The spatial probe test was used to detect changes within the spatial memory of the rats. This test was conducted on the second day after the place navigation test, during which time the platform was removed. The rats were placed into the water at a random entry point within the quadrant corresponding to the platform. The rats were allowed to swim freely in the water for 90 s. Then, we recorded the time that rats stayed in the quadrant corresponding to the platform.

### 2.4 Tissue preparation and hematoxylin–eosin (HE) staining

Chloral hydrate was used to anesthetize animals at a dose of 300 mg/kg. After fixation, the brains were removed, and then the rats immediately were euthanized with an injection of sodium pentobarbital at a dose of 150 mg. When the rat’s breathing stops and the heart stops beating, it is determined to be dead. The brain was dehydrated by gradient alcohol, and embedded in paraffin for immunohistochemical analysis, HE staining (HE, Wanleibio, China), and immunofluorescence. The paraffin-embedded tissue was cut into 3 µm-thick sections and paraffin was removed by gradient ethanol. Brain sections were stained with hematoxylin for 1–2 min and eosin for 30–60 s. After that, the sections were dehydrated with ethanol, sealed with neutral gum, and cleared with xylene [17]. After finding the hippocampus area under the low power microscope, we randomly selected five high power fields and counted the number of neurons in each high power field with Image J.

### 2.5 Immunohistochemistry and immunofluorescent staining

Sections of paraffin-embedded brain slices were deparaffinized and rehydrated before blocking non-specific antigen binding with 5% bovine serum albumin (Boster Biological Technology, China). The following primary antibodies were used: NPTX-1 (1:50; Abcam, Cambridge, UK), NPTX-2 (1:200; Abcam, Cambridge, UK), and CRP (1:100; Abcam, Cambridge, UK). Goat anti-rat/rabbit (Beijing Zhongshan Jinqiao Biotechnology, China) polymer enhancer and goat-resistant rabbit/rat IgG-conjugated polymer were added to each tissue section. Afterward, the immunostained sections were developed in diaminobenzidine (Beijing Zhongshan Jinqiao Biotechnology, China) and counterstained with a weak solution of hematoxylin solution. The stained tissue slices were visualized on a microscope (DMI6000B, Leica, USA).

The immunofluorescence staining protocol on the first day was the same as the immunohistochemical staining protocol, which was performed to evaluate the localization of *NPTX-1*, *NPT*X-2, and *CRP*. The luciferin antibody (100 µL) was added to each stained section. After incubating the sections in the dark at room temperature for 1 h, a drop of 4′,6-diamindino-2-phenylindole (Beijing Zhongshan Jinqiao Biotechnology, China) staining agent was added to each tissue section. An anti-quenching agent was added to seal the sections. The staining results were observed under a fluorescence microscope.

### 2.6 Western blot

The radioimmunoprecipitation assay buffer was used to isolate the total protein, and then it was quantified using bicinchoninic acid assay (Thermo Invitrogen, Waltham, MA). The total protein (20 μg) was added into every well, and then transferred onto polyvinylidene fluoride membrane (IPVH00010, Millipore, USA). The primary antibody including Bax (1:1,000, Proteintech), caspase-3 (1:1,000, Proteintech), and actin (1:1,000, Proteintech) were incubated with membrane at 4°C overnight. Secondary goat anti-rabbit antibody (1:5,000, Jackson ImmunoResearch, USA) and goat anti-mouse IgG (1:5,000, Jackson ImmunoResearch, USA) were incubated with membrane at room temperature for 2 h. The protein bands were visualized and the images were analyzed through chemiluminescence meter (4600, Tanon, Shanghai, China).

### 2.7 Statistical analysis

The experimental data positioning navigation and space exploration time from MWM were recorded and analyzed using one-way ANOVA method in SPSS software 22.0 (SPSS Inc., Chicago, IL, USA). The data were exhibited as mean ± standard deviation. Then, the immunohistochemical staining results of NPTX-1, NPTX-2, and CRP were semi-quantitatively analyzed, and ten fields of view in and around the hippocampus were selected for high-magnification imaging through an optical microscope, and the integrated optical density (IOD) value of each image was measured using ImageJ digital software. The average value of the 10 IOD values obtained for each slice represents the average IOD value of the slice. The IOD values obtained from the brain slices of mice in different groups are expressed as mean ± SD. Meanwhile, the immunofluorescence results to evaluate the gene localization were measured using ImageJ digital software. The formula was as follows: average fluorescence intensity (Mean) = sum of fluorescence intensity in this area (IntDen)/area of this area (Area). The difference between the groups was analyzed using one-way analysis of variance. A *P* value <0.05 was deemed statistically significant.


**Ethical approval:** The research related to animals’ use has been complied with all the relevant national regulations and institutional policies for the care and use of animals. Approval was obtained from the Ethics Committee of The First Hospital of Harbin Medical University (Approval No. SYXK (black) 2017-003) and conducted in strict accordance to the standard of the Guide for the Care and Use of Laboratory Animals published by the Ministry of Science and Technology of the People’s Republic of China in 2006.

## 3 Results

### 3.1 MWM test

**Figure 1:**
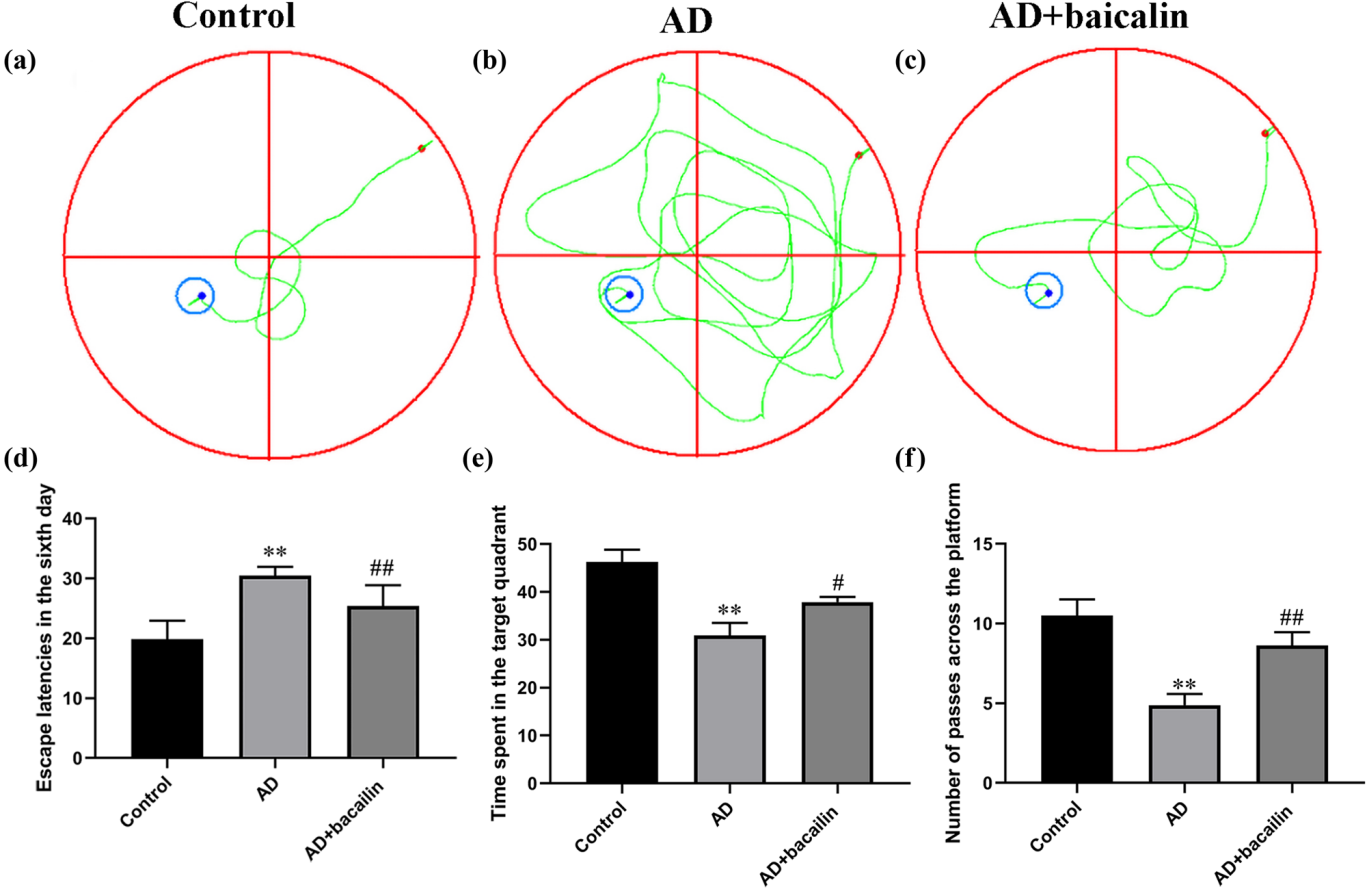
The swimming paths of the MWM test of each group: (a) control group, (b) AD group, (c) AD + baicalin group, (d) escape latencies on the sixth day, (e) time spent in the target quadrant, and (f) number of passes across the platform. Abbreviation: AD, Alzheimer’s disease.

The MWM test was used to assess the memory and spatial learning of rats. It is on the sixth day that the memory and spatial learning of rats was improved in AD + baicalin group when compared with AD group (Figure 1a–c). In addition, as shown in Table 1, with the extension of training days, the time required by different groups of rats to find the platform showed gradually different travel characteristics. On the sixth day, the escape latencies of the AD + baicalin group rats were significantly shorter than those from rats in the AD group (Figure 1d). In addition, we used a space exploration experiment to investigate whether the spatial memory of rats changed. We observed and recorded the time the rats spend in the target quadrant of the platform as well as the number of times the animals crossed the platform. As indicated in Figure 1 and f, baicalin treatment could significantly improve the number of times the animals crossed the platform and time spent in the target quadrant of the platform (Tables 2 and 3). These results indicated that baicalin could improve the memory and spatial learning of AD rats.

**Table 1:** Escape latencies in MWM training trials in different groups of everyday

Training days (*n*)	Grouping	The number of cases/group	Mean ± SD (s)	*P* value
1	The control group	10	87.122 ± 1.977	
	The AD model group	10	88.415 ± 2.905	0.392
	The baicalin group	10	86.827 ± 3.345	0.857; 0.401
2	The control group	10	73.299 ± 2.578	
	The AD model group	10	77.085 ± 3.355	0.055
	The baicalin group	10	76.597 ± 2.954	0.067; 0.795
3	The control group	10	67.236 ± 3.751	
	The AD model group	10	70.868 ± 2.619	0.084
	The baicalin group	10	71.091 ± 4.905	0.159; 0.924
4	The control group	10	45.111 ± 4.730	
	The AD model group	10	51.786 ± 4.538	0.032*
	The baicalin group	10	48.136 ± 2.650	0.210; 0.127
5	The control group	10	27.705 ± 6.038	
	The AD model group	10	45.489 ± 4.305	**0.000** ^*^
	The baicalin group	10	38.509 ± 3.473	**0.005** ^*^ **; 0.012** ^**#**^
6	The control group	10	19.852 ± 3.087	
	The AD model group	10	30.478 ± 1.465	**0.000** ^*^
	The baicalin group	10	25.413 ± 3.456	**0.015** ^*^; **0.008** ^**#**^

*n*: number; s: second; SD: standard deviation. *Compared with control group; #compared with AD model group. Note: Bold indicates statistical significance.

**Table 2:** Time spent in the target quadrant of different mouse in each group

Grouping	The number of cases/group	Mean ± SD (s)	*P* value
The control group	10	46.267 ± 8.075	
The AD model group	10	30.879 ± 8.359	**0.009** ^*^
The baicalin group	10	37.862 ± 3.429	**0.041** ^*^; **0.015** ^**#**^

*Compared with control group; #compared with model group. Note: Bold indicates statistical significance.

**Table 3:** Number of passes across the platform of different mouse in each group

Grouping	The number of cases/group	Mean ± SD (s)	*P* value
The control group	10	10.500 ± 3.207	
The AD model group	10	4.875 ± 2.231	**0.001** ^*^
The bacialin group	10	8.625 ± 2.615	**0.221**; **0.008** ^**#**^

*Compared with control group; #compared with model group. Note: Bold indicates statistical significance.

### 3.2 Effect of baicalin on neuronal cell numbers of AD rats

**Figure 2:**
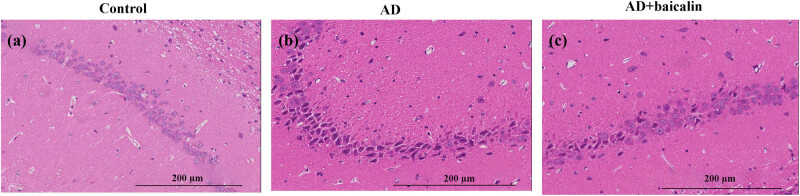
Effect of baicalin on the number of hippocampal neurons: (a) HE staining in the control group, (b) HE staining in the AD group, and (c) HE staining in the AD + baicalin group. Abbreviation: HE, hematoxylin–eosin; AD, Alzheimer’s disease.

**Figure 3:**
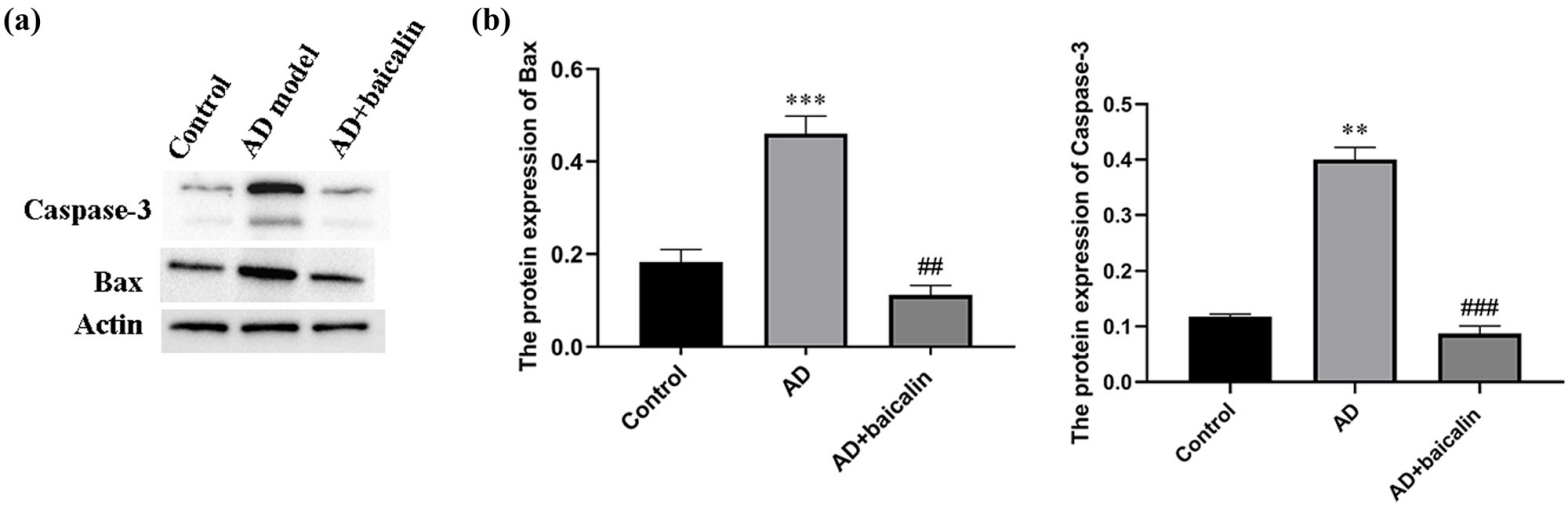
Effect of baicalin on neuronal apoptosis: (a) protein band and (b) gray-scale analysis of the protein band. **P < 0.01, ***P < 0.001, control vs AD model; ##*P* < 0.01, ###*P* < 0.001, AD model vs AD + baicalin. Abbreviation: AD, Alzheimer’s disease.

In the controls, no obvious cell deletion of the pyramidal cells was found. The morphology of the cells was complete, the nucleus was clear, and the cells were round or quasi-round (Figure 2a). However, in the AD group, the cell structures were observed to be disordered, the pyramidal cells were anomalous, and the cell membranes were narrowing. The nuclei of the damaged cells showed pyknosis, the nuclear membranes were not clear, and the nucleoli had disappeared (Figure 2b). The arrangement and morphology of cells in the AD + baicalin group was improved compared with the AD group (Figure 2c). The average number of hippocampal neurons in the AD group was significantly lower than that of the control group (2.896 × 10^4^ cell/mm^3^ vs 4.132 × 10^4^ cell/mm^3^, *P* < 0.05). However, the mean number of hippocampal neurons in the AD + baicalin group was significantly higher than the AD model group (3.498 × 10^4^ cell/mm^3^, *P* < 0.05) (Figure 2). Moreover, we validated the apoptosis of neurons through examining the expression of bax and caspase-3. As shown in Figure 3, the results indicated that baicalin could effectively inhibit the expression of bax and caspase-3 (*P* < 0.01; *P* < 0.001), which were consistent with the results of HE staining.

### 3.3 Effect of baicalin on NPTX-1, NPTX-2, and CRP expression of immunohistochemistry and immunofluorescence staining

**Figure 4:**
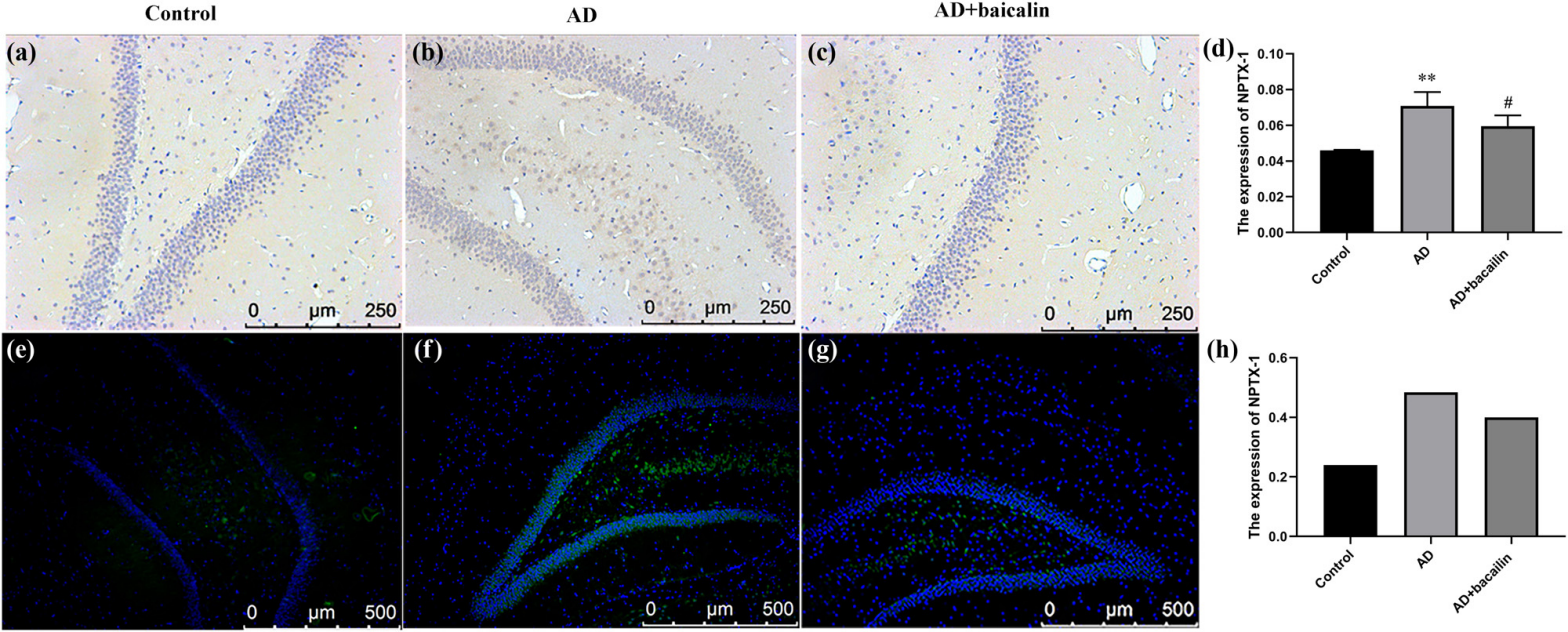
Effect of baicalin on NPTX-1 immunohistochemical (a–c) and immunofluorescence (d–f) staining: (a) immunohistochemical staining in the control group, (b) immunohistochemical staining in the AD group, (c) immunohistochemical staining in the AD + baicalin group, (d) quantification for immunohistochemical staining, (e) immunofluorescence in the control group, (f) immunofluorescence in the AD model group, (g) immunofluorescence in the AD + baicalin group, and (h) quantification for immunofluorescence. Abbreviation: AD: Alzheimer’s disease; NPTX-1: neuronal pentraxin-1.

The expression of NPTX-1, NPTX-2, and CRP in rats’ brain was examined using immunohistochemistry. Then, we evaluated the localization of NPTX-1, NPTX-2, and CRP using immunofluorescence staining. The results indicated that the expression of NPTX-1 in the AD + baicalin group was significantly reduced compared to the AD group (0.05953 ± 0.00603 vs 0.07076 ± 0.00788, *P* = 0.000) (Figure 4a–d). The fluorescence intensity in the AD model group was stronger than that of the controls and the AD + baicalin group (Figure 4e–h; Table 3).

**Figure 5:**
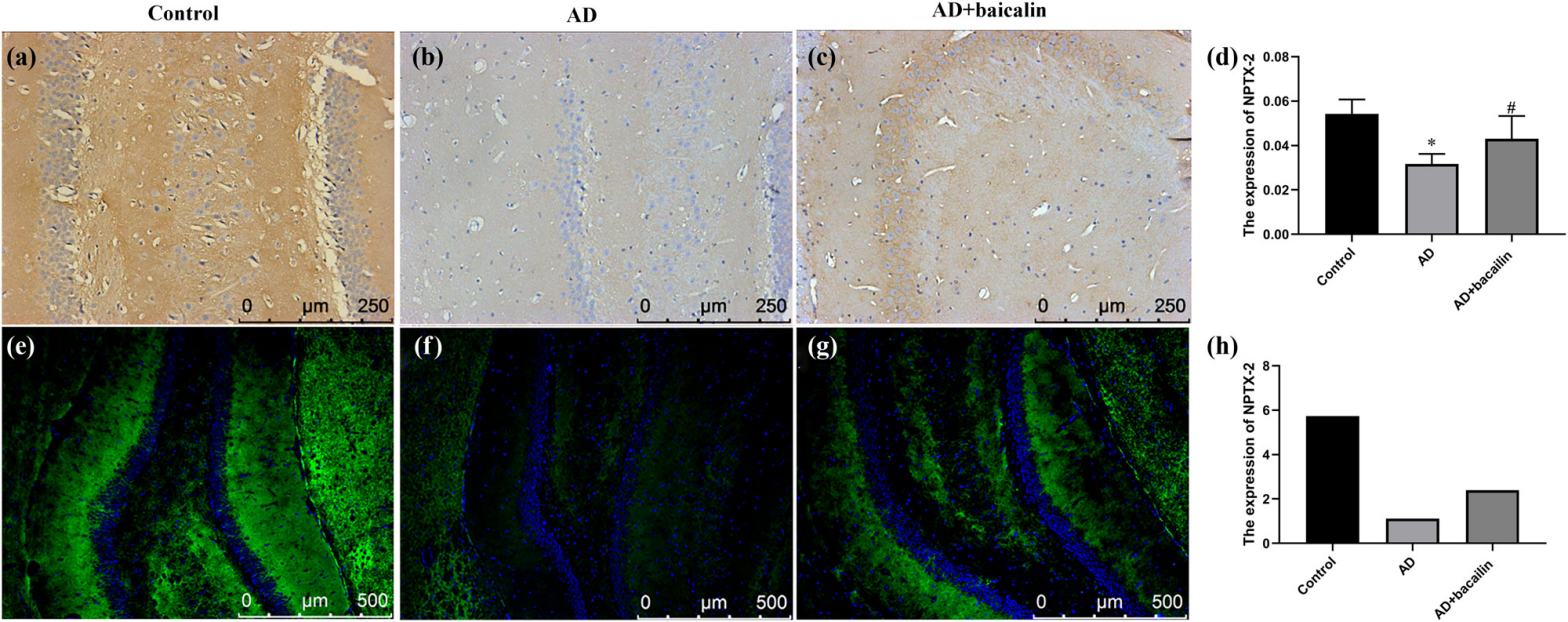
Effect of baicalin on NPTX-2 immunohistochemical (a–c) and immunofluorescence (d–f) staining: (a) immunohistochemical staining in the control group, (b) immunohistochemical staining in the AD model group, (c) immunohistochemical staining in the AD + baicalin group, (d) quantification for immunohistochemical staining, (e) immunofluorescence in the control group, (f) immunofluorescence in the AD model group, (g) immunofluorescence in the AD + baicalin group, and (h) quantification for immunofluorescence. Abbreviation: AD: Alzheimer’s disease; NPTX-2: neuronal pentraxin-2.

**Figure 6:**
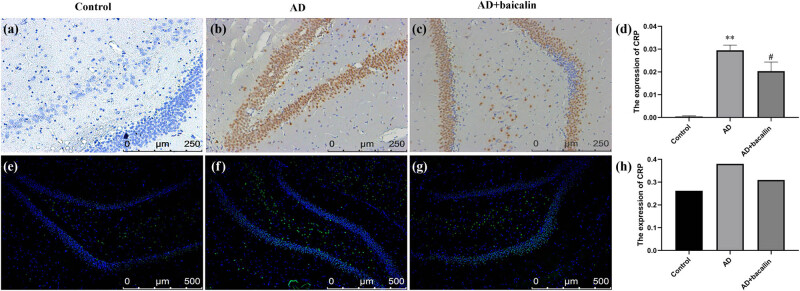
Effect of baicalin on CRP immunohistochemical (a–c) and immunofluorescence (d–f) staining: (a) immunohistochemical staining in the control group, (b) immunohistochemical staining in the AD model group, (c) immunohistochemical staining in the AD + baicalin group, (d) quantification for immunohistochemical staining, (e) immunofluorescence in the control group, (f) immunofluorescence in the AD model group, (g) immunofluorescence in the baicalin group, and (h) quantification for immunofluorescence. Abbreviation: AD: Alzheimer’s disease; NPTX-2: neuronal pentraxin-2; CRP: C-reaction protein.

Besides, the expression of NPTX-2 in the AD + baicalin group was significantly improved compared with the AD group (*P* = 0.033) (Figure 5a–d; Table 3). Meanwhile, the fluorescence intensity of the AD model group was stronger than that of the controls and the AD + baicalin group (Figure 5e–h). In addition, the expression of CRP in the AD + baicalin group (0.02034 ± 0.00397) was significantly reduced compared to the AD model group (*P* = 0.021) (Figure 6–d). The fluorescence intensity in the AD model group was stronger than that of the controls and the AD + baicalin group (Figure 5e–h; Table 4).

**Table 4:** Expression of NPTX-1, NPTX-2, and CRP in each group of different mouse’ brain tissue

*P* value	Grouping	The number of cases/group	Mean ± SD	*P* value
NPTX-1	The control group	10	0.00046 ± 0.00028	
	The AD model group	10	0.07076 ± 0.00788	**0.000** ^*^
	The baicalin group	10	0.05953 ± 0.00603	**0.000** ^*^ **; 0.020** ^**#**^
NPTX-2	The control group	10	0.05427 ± 0.006504	
	The AD model group	10	0.03168 ± 0.00453	**0.000** ^*^
	The baicalin group	10	0.04303 ± 0.01025	**0.047** ^*^ **; 0.033** ^**#**^
CRP	The control group	10	0.00046 ± 0.00028	
	The AD model group	10	0.02948 ± 0.00715	**0.000** ^*^
	The baicalin group	10	0.02034 ± 0.00397	**0.000** ^*^ **; 0.021** ^**#**^

SD: standard deviation. *Compared with control group; #compared with model group. Note: Bold indicates statistical significance.

## 4 Discussion

Previous studies demonstrated that baicalin and PTXs play the vital role in the development of AD. Previous studies did not show that baicalin impacts the expression of NPTX-1, NPTX-2, and CRP. Baicalin is a vital element of traditional Chinese, which can effectively protect against AD pathology [18]. However, the theoretical mechanism of baicalin affecting the expression of PTX is still unclear. Our results offer a basic theory for the regulation of NPTX-1, NPTX-2, and CRP expression by baicalin as well as the treatment of AD. In our study, the incubation period of AD rats treated with baicalin was significantly less than that of the untreated group. At the same time, the time spent in the target quadrant and the number of times to cross the platform has also been significantly improved. Moreover, baicalin is effective in preventing or curing Aβ-induced cognitive decline. Xiao et al. [19] demonstrated that baicalin shows a protective effect against Aβ25-35-induced learning and memory deficits. Ding et al. [20] and Xiong et al. [21] showed that baicalin availably improved Aβ-induced learning and memory deficit in AD model rats. Moreover, current studies have shown that injecting Aβ1-40 reduces the number of hippocampal neurons. Not surprisingly, when compared with the control group, the number of hippocampal neurons in the AD model group was significantly reduced. The hippocampus is one of the central areas associated with learning and memory, particularly short-term memory. Impaired short-term memory function is usually considered as a key symptom of AD [21]. The number of hippocampal neurons in the AD + baicalin group was significantly higher compared with the AD group. These results suggested that baicalin improves AD functional abnormalities and pathological changes in damaged rat models, and may play a certain role in delaying the development of AD.

Besides, NPTX-1 and NPTX-2 can participate in the formation, homeostasis, and plasticity of synaptic, and other processes alone or jointly, which play an important role in the process of clearing the synaptic debris generated during synaptic remodeling [22]. Both the two secreted NPTX1 and NPTX2 have been found in Aitana et al. study to be decreased in AD compared with controls and appear to be markers of disease progression in AD [23]. However, other studies suggested that NPTX-1 and NPTX-2 are usually secreted in the form of oligomeric complexes and exist in nervous tissue, but their proportion in oligomeric complexes depends on their participation in pathophysiological processes, neuronal activity, and neuronal state [24,25]. It is suggested that the interaction between NPTX-1 and NPTX-2 might be synergistic and have antagonistic effects. In this study, we found that the expression of NPTX-1 in the AD model group was significantly increased, while NPTX-2 expression was decreased. This result was similar to findings from a study by Abad et al. [7], which showed that NPTX-1 was increased in cellular processes surrounding amyloid plaques in the cerebral cortices and hippocampi of APP/PS1 transgenic mice. A study from Cummings et al. observed strong NPTX1 staining in the dystrophic neurites within the plaque [26]. Furthermore, the increasing expression of NPTX-1 in AD model might due to that NPTX-1 improved synaptic damage and activated the apoptosis of neuronal cells [27]. These results that the apoptosis protein including bax and caspase-3 were also highly expressed in AD model also confirmed this hypothesis. Moreover, the decreased expression of NPTX-2 in AD model was related to MTL atrophy and memory decline [28]. Notably, we found that baicalin could reverse the expression of NPTX-1 and NPTX-2 in AD. This study suggested that NPTX-1 and NPTX-2 are novel biomarkers in treating AD with baicalin. Further experiments need to be performed to verify the expression of NPTX-1 and NPTX-2 in AD and its function in therapy of baicalin.

Schmidt et al. [29] found that hemoglobin G-Honolulu-elevated plasma CRP levels in midlife are a risk factor for AD. Paradoxically, Nilsson et al. [30] and Yarchoan [31] found that patients with established AD expressed low plasma CRP levels. Our results are consistent with previous reports showing improved levels of CRP in AD, which indicate its potential function as a biomarker for the diagnosis of AD. At present, the relationship between the levels of CRP expression and AD is unclear, which encourages us to further investigate the role of CRP in the pathophysiology of AD.

## 5 Conclusion

In conclusion, baicalin may improve memory function and learning ability in a rat model of AD. We believe that the therapeutic actions of baicalin are linked with its ability to downregulate NPTX-1 and CPR, and upregulate NPTX-2 in a rat AD model.

## Abbreviations

AD: Alzheimer’s disease
CRP: C-reactive protein
NPTX: neuronal pentraxin
SAP: amyloid substance
SD: Sprague Dawley
SD: standard deviation

## References

[1] Scheltens P, De Strooper B, Kivipelto M, Holstege H, Chételat G, Teunissen CE, et al. Alzheimer’s disease. Lancet (London, Engl). 2021;397(10284):1577–90. 10.1016/S0140-6736(20)32205-4 PMC8354300 33667416

[2] Blanchard JW, Akay LA, Davila-Velderrain J, von Maydell D, Mathys H, Davidson SM, et al. APOE4 impairs myelination via cholesterol dysregulation in oligodendrocytes. Nature. 2022;611(7937):769–79. 10.1038/s41586-022-05439-w PMC9870060 36385529

[3] Bellenguez C, Küçükali F, Jansen IE, Kleineidam L, Moreno-Grau S, Amin N, et al. New insights into the genetic etiology of Alzheimer’s disease and related dementias. Nat Genet. 2022;54(4):412–36. 10.1038/s41588-022-01024-z PMC9005347 35379992

[4] Welsh KA, Butters N, Hughes JP, Mohs RC, Heyman A. Detection and staging of dementia in Alzheimer’s disease. Use of the neuropsychological measures developed for the consortium to establish a registry for Alzheimer’s disease. Arch Neurol. 1992;49(5):448–52. 10.1001/archneur.1992.00530290030008 1580805

[5] Selkoe DJ. Alzheimer’s disease is a synaptic failure. Sci (New York, NY). 2002;298(5594):789–91. 10.1126/science.1074069 12399581

[6] Ma QL, Teng E, Zuo X, Jones M, Teter B, Zhao EY, et al. Neuronal pentraxin 1: a synaptic-derived plasma biomarker in Alzheimer’s disease. Neurobiol Dis. 2018;114:120–8. 10.1016/j.nbd.2018.02.014 PMC8092920 29501530

[7] Abad MA, Enguita M, DeGregorio-Rocasolano N, Ferrer I, Trullas R. Neuronal pentraxin 1 contributes to the neuronal damage evoked by amyloid-beta and is overexpressed in dystrophic neurites in Alzheimer’s brain. J Neurosci. 2006;26(49):12735–47. 10.1523/JNEUROSCI.0575-06.2006 PMC6674827 17151277

[8] Koch SM, Ullian EM. Neuronal pentraxins mediate silent synapse conversion in the developing visual system. J Neurosci: Off J Soc Neurosci. 2010;30(15):5404–14. 10.1523/JNEUROSCI.4893-09.2010 PMC2885289 20392962

[9] Cheng O, Li Z, Han Y, Jiang Q, Yan Y, Cheng K. Baicalin improved the spatial learning ability of global ischemia/reperfusion rats by reducing hippocampal apoptosis. Brain Res. 2012;1470:111–8. 10.1016/j.brainres.2012.06.026 22796597

[10] Zhao J, Lu S, Yu H, Duan S, Zhao J. Baicalin and ginsenoside Rb1 promote the proliferation and differentiation of neural stem cells in Alzheimer’s disease model rats. Brain Res. 2018;1678:187–94. 10.1016/j.brainres.2017.10.003 29038007

[11] Zhou QB, Jin YL, Jia Q, Zhang Y, Li LY, Liu P, et al. Baicalin attenuates brain edema in a rat model of intracerebral hemorrhage. Inflammation. 2014;37(1):107–15. 10.1007/s10753-013-9717-9 PMC3929027 23974988

[12] Jin X, Liu MY, Zhang DF, Zhong X, Du K, Qian P, et al. Baicalin mitigates cognitive impairment and protects neurons from microglia-mediated neuroinflammation via suppressing NLRP3 inflammasomes and TLR4/NF-κB signaling pathway. CNS Neurosci Ther. 2019;25(5):575–90. 10.1111/cns.13086 PMC6488900 30676698

[13] Yin F, Liu J, Ji X, Wang Y, Zidichouski J, Zhang J. Baicalin prevents the production of hydrogen peroxide and oxidative stress induced by Aβ aggregation in SH-SY5Y cells. Neurosci Lett. 2011;496(2):76–9. 10.1016/j.neulet.2011.01.055 21276834

[14] Li J, Wang G, Zhang Y, Fan X, Yao M. Protective effects of baicalin against L-glutamate-induced oxidative damage in HT-22 cells by inhibiting NLRP3 inflammasome activation via Nrf2/HO-1 signaling. Iran J basic Med Sci. 2023;26(3):351–8. 10.22038/IJBMS.2023.64318.14149 PMC9922368 36865047

[15] Morris R. Developments of a water-maze procedure for studying spatial learning in the rat. J Neurosci Methods. 1984;11(1):47–60. 10.1016/0165-0270(84)90007-4 6471907

[16] Qi X-M, Wang C, Chu X-K, Li G, Ma J-F. Intraventricular infusion of clusterin ameliorated cognition and pathology in Tg6799 model of Alzheimer’s disease. BMC Neurosci. 2018;19(1):2. 10.1186/s12868-018-0402-7 PMC5785859 29370749

[17] Quan Q, Wang J, Li X, Wang Y. Ginsenoside Rg1 decreases Aβ(1-42) level by upregulating PPARγ and IDE expression in the hippocampus of a rat model of Alzheimer’s disease. PLoS ONE. 2013;8(3):e59155. 10.1371/journal.pone.0059155 PMC3592813 23520555

[18] Li Y, Zhuang P, Shen B, Zhang Y, Shen J. Baicalin promotes neuronal differentiation of neural stem/progenitor cells through modulating p-stat3 and bHLH family protein expression. Brain Res. 2012;1429:36–42. 10.1016/j.brainres.2011.10.030 22088824

[19] Xiao W, Cao XL, Zhang R, Gao CZ, Jing DU, Yin TZ, et al. Baicalin attenuates Aβ_(25-35) induced learning and memory disorders in mice and its possible mechanism. Chinese J Pharmacol Toxicol. 2017;31:59–63.

[20] Ding H, Wang H, Zhao Y, Sun D, Zhai X. Protective effects of baicalin on Aβ 1–42-induced learning and memory deficit, oxidative stress, and apoptosis in rat. Cell Mol Neurobiol. 2015;35(5):623–32. 10.1007/s10571-015-0156-z PMC11486265 25596671

[21] Xiong J, Wang C, Chen H, Hu Y, Tian L, Pan J, et al. Aβ-induced microglial cell activation is inhibited by baicalin through the JAK2/STAT3 signaling pathway. Int J Neurosci. 2014;124(8):609–20. 10.3109/00207454.2013.865027 24219385

[22] Lee SJ, Wei M, Zhang C, Maxeiner S, Pak C, Calado Botelho S, et al. Presynaptic neuronal pentraxin receptor organizes excitatory and inhibitory synapses. J Neurosci: Off J Soc Neurosci. 2017;37(5):1062–80. 10.1523/JNEUROSCI.2768-16.2016 PMC5296791 27986928

[23] Sogorb-Esteve A, Nilsson J, Swift IJ, Heller C, Bocchetta M, Russell LL, et al. Differential impairment of cerebrospinal fluid synaptic biomarkers in the genetic forms of frontotemporal dementia. Alzheimer’s Res Ther. 2022;14(1):118. 10.1186/s13195-022-01042-3 PMC9429339 36045450

[24] Swanson A, Wolf T, Sitzmann A, Willette AA. Neuroinflammation in Alzheimer’s disease: pleiotropic roles for cytokines and neuronal pentraxins. Behav Brain Res. 2018;347:49–56. 10.1016/j.bbr.2018.02.015 PMC5988985 29462653

[25] Bolsewig K, Hok AHYS, Sepe FN, Boonkamp L, Jacobs D, Bellomo G, et al. A combination of neurofilament light, glial fibrillary acidic protein, and neuronal pentraxin-2 discriminates between frontotemporal dementia and other dementias. J Alzheimer’s Dis: JAD. 2022;90(1):363–80. 10.3233/JAD-220318 PMC9661338 36120776

[26] Cummings DM, Benway TA, Ho H, Tedoldi A, Fernandes Freitas MM, Shahab L, et al. Neuronal and peripheral pentraxins modify glutamate release and may interact in blood–brain barrier failure. Cerebral Cortex (New York, NY: 1991). 2017;27(6):3437–48. 10.1093/cercor/bhx046 28334103

[27] Hudry E, Dashkoff J, Roe AD, Takeda S, Koffie RM, Hashimoto T, et al. Gene transfer of human Apoe isoforms results in differential modulation of amyloid deposition and neurotoxicity in mouse brain. Sci Transl Med. 2013;5(212):212ra161. 10.1126/scitranslmed.3007000 PMC4334150 24259049

[28] Swanson A, Willette AA. Initiative AsDN. Neuronal Pentraxin 2 predicts medial temporal atrophy and memory decline across the Alzheimer’s disease spectrum. Brain Behav Immun. 2016;58:201–8. 10.1016/j.bbi.2016.07.148 PMC5349324 27444967

[29] Schmidt R, Schmidt H, Curb JD, Masaki K, White LR, Launer L. Early inflammation and dementia: a 25-year follow-up of the Honolulu–Asia aging study. Ann Neurol. 2002;52(2):168–74. 10.1002/ana.10265 12210786

[30] Nilsson K, Gustafson L, Hultberg B. C-reactive protein: vascular risk marker in elderly patients with mental illness. Dement Geriatr Cogn Disord. 2008;26(3):251–6. 10.1159/000160957 18841009

[31] Yarchoan M, Louneva N, Xie SX, Swenson FJ, Hu W, Soares H, et al. Association of plasma C-reactive protein levels with the diagnosis of Alzheimer’s disease. J Neurologic Sci. 2013;333(1–2):9–12. 10.1016/j.jns.2013.05.028 PMC3815534 23978419

